# Optimal contact definition for reconstruction of Contact Maps

**DOI:** 10.1186/1471-2105-11-283

**Published:** 2010-05-27

**Authors:** Jose M Duarte, Rajagopal Sathyapriya, Henning Stehr, Ioannis Filippis, Michael Lappe

**Affiliations:** 1 Max Planck Institute for Molecular Genetics, Ihnestr. 63-73, 14195 Berlin, Germany; 2 Laboratory of Biomolecular Research, Paul Scherer Institut, 5232 Villigen PSI, Switzerland; 3 Centre for Bioinformatics, Division of Molecular Biosciences, Imperial College London, London SW7 2AZ, UK

## Abstract

**Background:**

Contact maps have been extensively used as a simplified representation of protein structures. They capture most important features of a protein's fold, being preferred by a number of researchers for the description and study of protein structures. Inspired by the model's simplicity many groups have dedicated a considerable amount of effort towards contact prediction as a proxy for protein structure prediction. However a contact map's biological interest is subject to the availability of reliable methods for the 3-dimensional reconstruction of the structure.

**Results:**

We use an implementation of the well-known distance geometry protocol to build realistic protein 3-dimensional models from contact maps, performing an extensive exploration of many of the parameters involved in the reconstruction process. We try to address the questions: a) to what accuracy does a contact map represent its corresponding 3D structure, b) what is the best contact map representation with regard to reconstructability and c) what is the effect of partial or inaccurate contact information on the 3D structure recovery. Our results suggest that contact maps derived from the application of a distance cutoff of 9 to 11Å around the *C*_*β *_atoms constitute the most accurate representation of the 3D structure. The reconstruction process does not provide a single solution to the problem but rather an ensemble of conformations that are within 2Å RMSD of the crystal structure and with lower values for the pairwise average ensemble RMSD. Interestingly it is still possible to recover a structure with partial contact information, although wrong contacts can lead to dramatic loss in reconstruction fidelity.

**Conclusions:**

Thus contact maps represent a valid approximation to the structures with an accuracy comparable to that of experimental methods. The optimal contact definitions constitute key guidelines for methods based on contact maps such as structure prediction through contacts and structural alignments based on maximum contact map overlap.

## Background

For over 30 years [1,2] contact maps have been used as an alternative representation of protein structures. A contact map is a 2-dimensional representation of the residue interactions in a protein structure. This 2-dimensional representation takes the form of a binary matrix. A given cell (i, j) of the matrix can only take two values, 1 if the residues i and j are in contact or 0 otherwise. The definition of interaction varies but it is usually based on some cut-off distance between the atoms of the two residues. One can also see this description from another perspective as a residue interaction graph (RIG) with residues as nodes and the contacts as edges. In this view the binary matrix is no more than the adjacency matrix representing the graph.

Although they constitute a simple 2-dimensional representation of the molecule, contact maps still capture all important features of a protein fold. As such they are an invaluable tool for the analysis of biological macromolecules. They provide a computationally tractable representation of an otherwise complex problem, with the important advantage of being structural descriptors independent of the coordinate frame. Thus providing a sort of internal coordinates description, rotationally and translationally independent. However the simplified representation loses on accuracy as compared to the original 3-dimensional model. Multiple applications can be found in the literature that make use of the concept. Contact maps have been used for development of structural alignment algorithms [3,4], for automatic domain identification [5,6], in structural modelling by the extraction of contact-based empirical potentials [7-10] or for the identification of residues critical for folding [11], stability [12] and function [13]. Furthermore they have been used as a proxy for 3-dimensional structure prediction by means of machine learning techniques in order to predict residue contacts from sequence information [14-18].

Several methods have been proposed in the past for the reconstruction of contact maps. Most of them develop around the common mathematical theory of distance geometry first applied to chemistry by Blumenthal [19]. The theory took really off when Crippen and Havel [20] applied it to the problem of protein structure determination by NMR methods. In a typical NMR experiment distances between spatially close Hydrogen atoms can be determined for a protein in solution through the detection of the Nuclear Overhausser Effect (NOE) [21]. The NOE data can be seen then as a set of distance ranges between some pairs of Hydrogen atoms. Distance geometry deals with distances between points and their embedding in 3-dimensional space. In principle given a proper metric matrix with all exact distances among a set of points an analytical solution to the embedding can be found easily. The problem becomes more complicated when not all distances are given (sparse distance map) and when only distance ranges rather than exact distances are known. This is the case of the NMR experiments and equivalently of contact maps: we know some distance ranges between pairs of atoms for which we would like to find 3-dimensional coordinates. A heuristic algorithm (named EMBED) to solve the problem was proposed by Crippen and Havel and has been applied extensively ever since. Other algorithms have been proposed such as the alternating projection algorithm by Glunt et al. [22] or the geometric build-up algorithm by Wu and Wu [23].

However the problem of reconstructability of protein contact maps has not been fully addressed in the literature. A few studies [24-26] have tried to evaluate the accuracy of the existing methods but they all lack in completeness of the test set and thorough assessment of the different parameters or do not provide fully realistic protein models but only *C*_*α *_traces.

**Figure 1:**
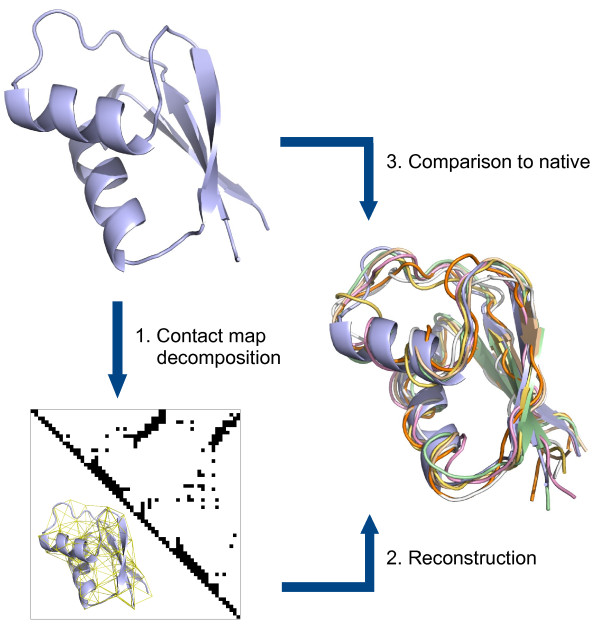
**Schematic representation of the optimization procedure**. 1) the native structure is decomposed into contact maps based on different definitions, 2) the 3D structure is reconstructed from contact information only, obtaining an ensemble of conformations, 3) the accuracy is measured against the original structure. The protein shown is PDB structure 1bxyA. The ensemble corresponds to 6 reconstructions (ribbon representation) in different colours and also contains the native protein (cartoon representation) in blue.

Our aim here is twofold. We would like to find what is the reconstruction accuracy for an average protein so that the limits of the utility of contact maps in protein structure prediction can be precisely assessed. As a second aim we are looking for optimal criteria in the definition of a contact map decomposition model: atoms selected as interaction centres and distance cut-off. By decomposing a representative set of PDB protein structures into residue interaction graphs and then reconstructing them based purely on the contact information we should be able to assess the accuracy and loss of information in the decomposition process by comparing to the original native structure (see Figure 1). If a specific contact map model that reconstructs optimally can be found, that would help direct efforts in prediction of contact maps. Previous work has looked at optimality of contact definition from very different points of view, mainly in relation to how well contacting pairs describe the residue propensities when discriminating decoys from native structures. Here we look at it in a purely geometrical way, we are intending to find out how much of the 3D geometrical topology is captured by the network of contacts. Additionally by introducing artificial noise in the contact maps we also look at the effect of inaccurate contact information in the 3-dimensional recovery, essential to the applicability of contacts for predictive purposes.

## Results and Discussion

We studied the reconstructability of a set of representative native PDB protein structures (see Methods). Firstly we decomposed the native proteins into contact maps with different contact type definitions and for several distance cut-offs. Then we used our reconstruction software to recreate the 3D structures based solely in the information supplied by the contact maps.

To measure the accuracy we then proceed by evaluating the RMSD of the generated models with the original structure. We measured the RMSD on the *C*_*α *_atoms over all residues, independent of whether the reconstructions were based on *C*_*α *_contact maps or not. This seems to be a well-established way of measuring the similarity between two structures especially when they are closely related and should facilitate the comparison to other published work. Another well-established method for structure comparison, GDT [27], was not deemed to be appropriate here as it is most useful in comparing structures over a broader range of dissimilarity as is the case in the CASP experiment.

### Optimal cut-off

**Figure 2:**
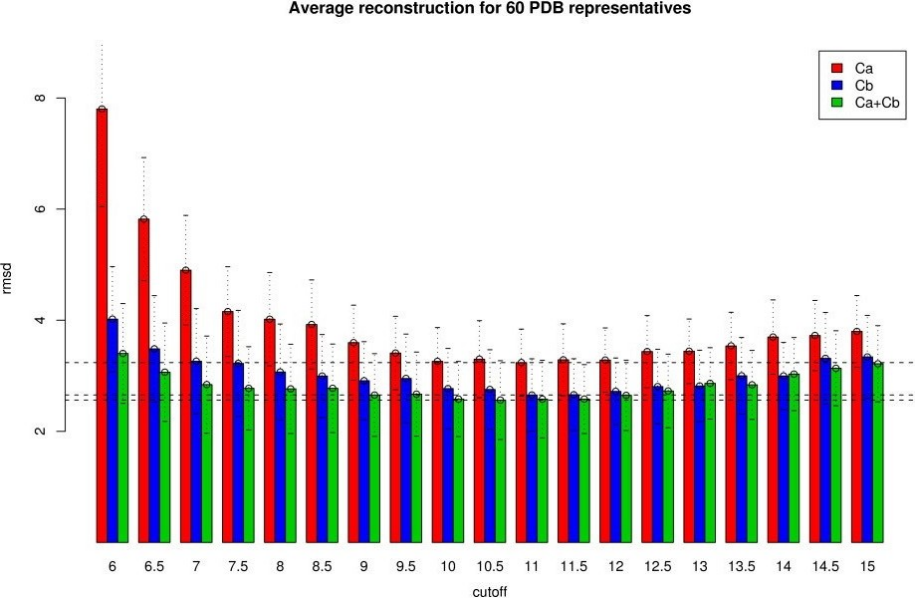
**Accuracy of reconstructions**. Reconstruction *C*_*α *_RMSD vs. distance cutoff for each of the contact definitions. Plotted are the mean accuracy values for the set of 60 proteins for *C*_*α*_, *C*_*β *_and *C*_*α *_+ *C*_*β *_contact definitions. Horizontal lines mark the minimum RMSD for each of them. The error bars represent the standard deviation across the distribution of 60 proteins.

In Figure 2 we present the accuracy of reconstruction as measured by RMSD vs. the distance cut-off for contact maps based on *C*_*α*_, *C*_*β *_and *C*_*α *_+ *C*_*β *_contact-types (see Methods for contact-type definitions).

The range of cut-offs chosen was based on values previously used in the literature keeping them within a biochemically sensible range: the minimum cut-off was 6Å as values below result in too sparse contact maps. At the other end we chose 15Å since beyond that the contact map starts to lose in information content becoming fully connected.

**Figure 3:**
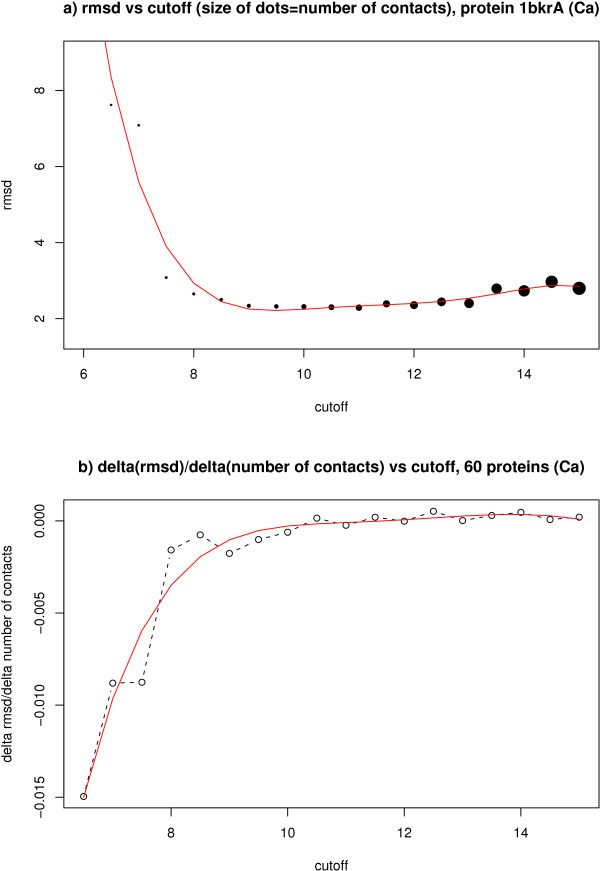
**Number of contacts and reconstruction accuracy**. a) RMSD values for the protein 1bkrA using *C*_*α *_as contact definition, the size of the dots represent the total number of contacts in the contact map for a particular cutoff. The red curve is a linear fit to a polynomial. b) RMSD delta over delta of number of contacts against the cut-off for *C*_*α *_contact definition for the average of the 60 proteins in the data set. The red curve is again a linear fit to a polynomial.

The first interesting observation is the existence of an optimal cut-off for all the contact types. This optimal value is not very precisely defined in most cases, it seems to span the cut-off distances from 9 to 11Å with higher cut-offs having only a marginal loss of accuracy. However we consider of a more significant value the lower cut-offs. First of all because of the biochemical meaning of the contacts. It is in the region about the 8Å cut-off where our definition of contact lead to distances between atoms that are in the range of the Van der Waals interactions. Also the information content of the contacts should be taken into account. As shown in Figure 3a the practically unchanged accuracy values in the higher cut-off regions are accompanied by an increase in the total number of contacts (the number of contacts increases roughly linearly with the distance cut-off). Thus we could see this as a loss of information content per contact i.e. we are adding a lot more information that is simply redundant. Figure 3b illustrates this better by representing the gain in accuracy with respect to contacts added vs the distance cut-off. The accuracy gain occurs only up to 8Å, after that there is no change as more contacts are added.

**Figure 4:**
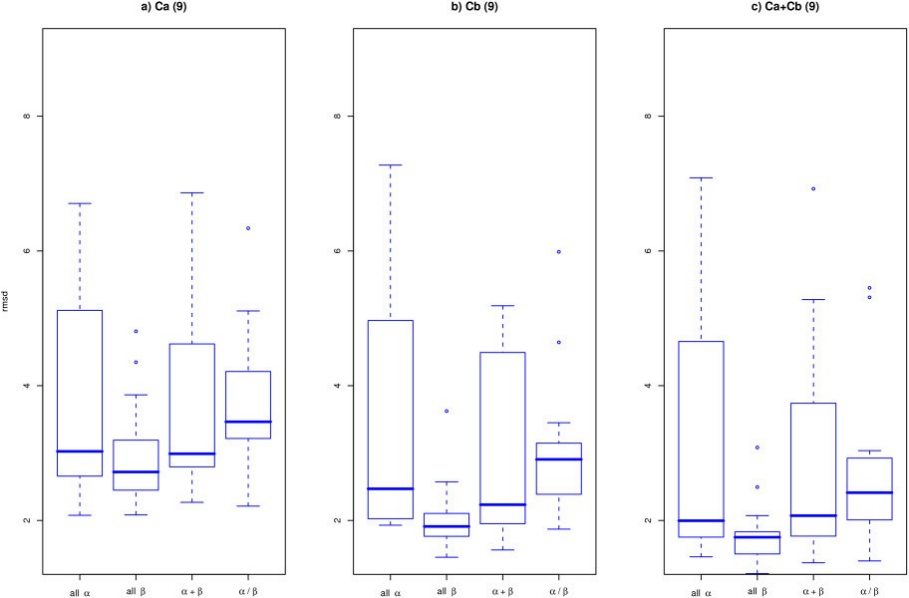
**Variability for different SCOP classes**. Reconstruction accuracy comparison for proteins in the four SCOP classes, using boxplots to depict the distributions of RMSD values. There are exactly 15 proteins per class from the set of 60 PDB representatives. a) For *C*_*α *_b) for *C*_*β *_and c) *C*_*α *_+ *C*_*β*_, all three at 9Å cutoff.

**Figure 5:**
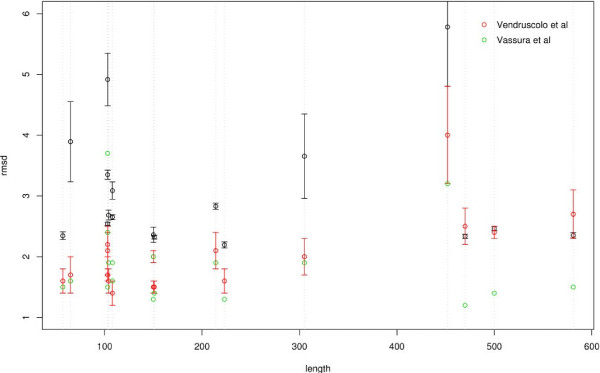
**Comparison to previous studies**. Comparison of our reconstruction RMSD values (black) with those of Vassura et al. (green) and Vendruscolo et al (red). The set is the one used by Vendruscolo and subsequently by Vassura. Two proteins were eliminated from their set because of ambiguities with the data. The error bars are for the variability across different runs (not reported by Vassura).

Additionally no dependence on the protein length across all cut-offs could be observed (see Figure 5). The reconstruction process seems to work with the same accuracy as measured by RMSD regardless of the protein size. This holds across all proteins tested (data not shown) and is in agreement with what similar studies found [26,24].

Our RMSD vs distance cut-off plots show no further improvement in accuracy beyond the optimal cut-off region. This is in clear disagreement with [26] where the reconstruction quality is reported to further increase for cut-off values as big as 18Å. This can be explained by the fundamentally different procedure of computing the reconstructed models: in our case an all atom approach with realistic regularization of the coordinates through a restraint-only harmonic potential was used for the construction of the models.

Vassura et al. on the other hand uses a simpler *C*_*α *_trace model, without a final refinement phase. Optimal threshold values found here are in agreement to some of the reported optimal values found in other studies. There has been many attempts in the past to find an optimal contact map definition with respect to both distance cut-off and interaction centre. The optimizations were based in different criteria according to what the focus was in the particular study.

Some authors like Gromiha et al. [28] studied the correlation of relative contact order with folding rate, finding that from several cut-offs 8Å gave the best correlations for the *C*_*α *_contact type when considering long range interactions only.

Karchin et al. [7] found that residue burial expressed as contact counts performs best at fold recognition for *C*_*β *_contact type with a cut-off of 14Å. Similarly Benkert et al. [8] used the same residue burial measure and surprisingly found that a cut-off of 9Å was optimal, possibly due to differences in normalisation procedures. Quite a few studies tried to find an optimal contact definition based on the discriminatory power of contact-based empirical potentials in distinguishing decoys from native structures. Bolser et al. [9] found that the best performing two-body potential was that derived from *C*_*β *_contact definition with a 12Å cut-off. Vendruscolo et al. [29] found that for the *C*_*α *_contact type the best cut-off was at 8.5Å for a two-body contact potential.

As contact maps are only meaningful in the context of obtaining 3D protein models the reconstructability criterium should not be neglected when considering a contact definition for instance in the prediction of contacts. Contacts containing more geometrical information will be more valuable when building 3-dimensional models. This is of special importance if we consider that the reconstruction of contact maps seems to be possible even with sparser contact maps (see [30,31]), which means that contacts even at optimal definitions still seem to contain redundant information.

### Optimal interaction centre

Comparing the accuracy values between the *C*_*α*_, *C*_*β *_and *C*_*α *_+ *C*_*β *_cases (see Figure 2) it is apparent that *C*_*α *_+ *C*_*β *_performs better across the whole range of cut-offs tested, with *C*_*β *_alone doing also better than *C*_*α*_. Figure 4 shows again this comparison for proteins divided into their respective SCOP classes. The trend holds within each of the SCOP classes.

Melo et al. [10] studying distance dependent empirical potentials explored several interaction centres concluding that the *C*_*β *_atom was the best performing atom centre. This seems to be a widely accepted result as indicates the use of the *C*_*β *_contact type for the contact prediction category at the Critical Assessment of protein Structure Prediction (CASP) experiment [32].

Our study, purely based on the 3D geometrical information content of the contacts, confirms the preference for *C*_*β *_as the interaction centre of choice. It seems natural that *C*_*β *_is better in order to derive empirical potentials as it spans both the backbone and the side-chain. But also it is a superior point of choice for embedding a 3D structure from interatomic distance restraints. The interaction centre is able to capture geometrical information for the backbone positioning as well as for the orientation of the side-chain leading to a more precise 3D description.

Also of interest is the fact that the combination of both *C*_*α *_and *C*_*β *_contacts leads still to better reconstruction performance, indicating that there is some more backbone information not contained in the *C*_*β *_restraints. This suggests an approach in the homology modelling of proteins based on distance restraints (see [33-35]): using two atoms per residue to restrain the geometry will lead to more precise models. We also obtained better accuracy results (data not shown) by choosing a backbone atom and a side-chain atom farther away from the *C*_*β*_.

### Reconstructions for different SCOP classes

We then address the question of whether the reconstruction process is dependant of the type of protein. In order to do so we separate our 60 proteins into the four SCOP classes to which they belong to, each of the classes containing 15 structures. Figure 4 shows the accuracy values for each of these four classes. The results hold for other cutoffs. It is striking that the accuracy and spread of the all-*β *group is significantly better than that of the other three. Interestingly the median values are not very far away for the 4 classes but the variances are hugely different especially for the all-*beta *case. Contrary to this result, in a similar study Saitoh et al. [24] stated that they did not encounter a dependency of the accuracy of reconstruction based on the SCOP class. This might be explained by the much smaller test set used in that study, 11 proteins in total and only 2 in the all-*β *class. Vassura et al. [26] did find some differences across different classes especially a lower accuracy for the all-*a *class, which we also observe here.

### Variability of the reconstruction ensembles

The reconstruction process inherently leads to a non-unique solution fully matching the contact map. We studied the variance of the ensemble of reconstructed structures. The average spread of the pairwise RMSD among the ensemble structures is in most cases below 2Å. In Table 1 we present the spread values for a 12 proteins subset (see Methods). An example ensemble can be seen in Figure 1.

**Table 1:** RMSD of reconstruction ensembles.

PDB code	SCOP class	Length	Ensemble's average RMSD
1bkrA	all-α	109	1.93
1oddA	all-α	118	2.76
1cemA	all-α	363	1.69
			
1pzcA	all-*β*	123	1.52
1onlA	all-*β*	128	1.67
1eurA	all-*β*	365	2.49
			
1e6kA	α/*β*	130	1.91
1o8wA	α/*β*	146	1.71
1edeA	α/*β*	310	1.62
			
1r9hA	α + *β*	135	3.11
1ugmA	α + *β*	125	2.17
1iu4A	α + *β*	331	3.70

The 12 proteins subset with chain lengths and the average pairwise RMSD of the reconstruction ensembles, based on *C*_*β *_contact maps with 8Å cut-off.

As seen in Figure 1 the reconstruction ensemble is reminiscent of an NMR structure ensemble, not surprisingly as both are based on fitting 3D coordinates to distance restraints. This shows another advantage of the contact map representation, namely that the conformational flexibility of the molecules is implicit in the model.

### Comparison to previous studies

For completeness of this work we compare our results to those of two previously published reconstruction methods [26,25]. In Figure 5 we present our results (black) for the set of 17 proteins used by Vendruscolo et al. and subsequently by Vassura et al. together with their results (red and green respectively). Our RMSD values are higher in most cases. Remarkably the values of Vassura et al. are a lot lower. However caution should be taken in this comparison as they do not report on the variability (error) of the result. As their algorithm (like the others) is stochastic the evaluation of the variability across different runs is important to consider. Another important issue to take into account is that these two previous studies are using a simpler representation of proteins, namely one based on only the *C*_*α *_atoms. In contrast here we are constructing full atom protein chains with realistic bonds and angles. This leads to higher RMSD values as more geometrical constraints need to be fulfilled.

### Tolerance to missing contacts and noise

As a final part of the study we then address the question of reconstruction of contact maps in the more realistic scenario of incomplete or noisy maps, which is likely to be the case when the input is a predicted set of contacts. To do this instead of using real predictions, for instance from homology or machine learning methods, we simulate incomplete and noisy contact maps to thoroughly explore the effect of noise in the process of reconstruction.

**Figure 6:**
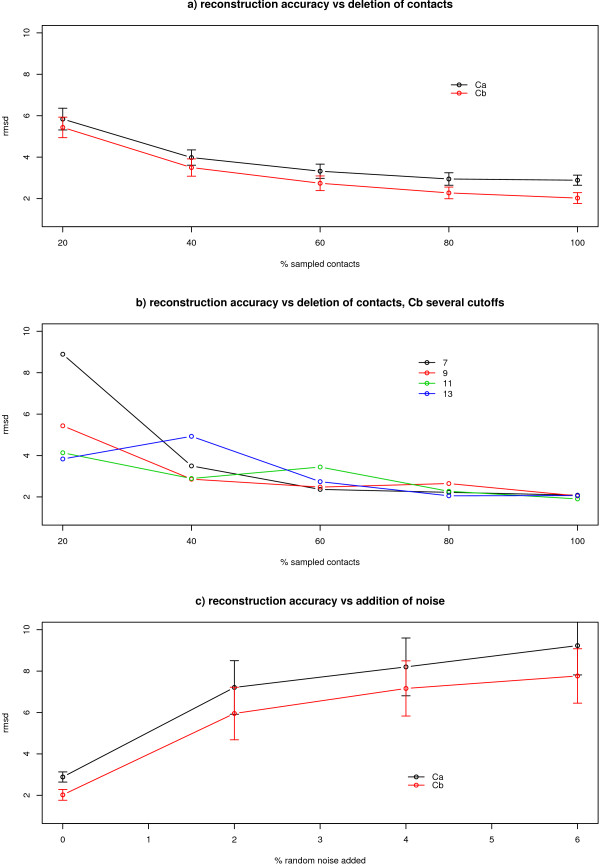
**Reconstruction for incomplete or noisy maps**. Behaviour of the reconstruction algorithm with noise or incomplete data. a) random subsets are sampled for *C*_*α *_and *C*_*β *_maps, b) random subsets are sampled for *C*_*β *_maps at different cut-offs (7, 9, 11 and 13, with different colours) and c) random contact noise is added to the map (*C*_*α *_and *C*_*β *_maps). The 12 proteins subset (see Methods) was used for this analysis. For each of the levels of noise 10 random samples were taken and 30 models generated. The variability within the different proteins in the set is represented with the error bars.

Figure 6a presents the reconstruction accuracy versus the percentage of contact deletion. Thus we are simulating a prediction that misses contacts but with a 100% precision. The striking observation here is that the reconstruction seems to be very robust to missing information, thus indicating that there is a lot of redundancy in the contacts. A previous study in our group [30] deals with this problem in more depth and finds that one can even predict rationally a subset of contacts that somehow contain the most structural information.

Interestingly enough there seems to be a non-linear relationship in the information redundancy with respect to cut-off. Figure 6b represents as before the reconstruction RMSD versus the deletion of contacts but this time only for contact type *C*_*β *_and different cut-offs. The loss of accuracy with lower percentage sampled subsets seems to decrease with higher cut-offs. Thus for the same percentage deletion one can recreate the original structure better with contact maps of higher cut-offs, i.e. the redundancy is higher. The second test that we perform intends to asses the robustness of the 3D recovery process with respect to the presence of noise, the case of a more realistic prediction with false positives. Figure 6c represents the reconstruction accuracy versus the percentage of noise added. The behaviour here is totally different than before. An addition of only 2% of random contacts severely affects the 3D recovery process. The *C*_*β *_definition behaves better at all levels of noise.

An existing application [36] is reported to perform better with noisy contact maps, but this seems to be due to their pre-filtering based on finding well connected nodes, equivalent to finding contact clusters. As the test is against randomly added contacts this is not a very realistic filtering. In a real scenario a) one would not have all well-connected real contacts of the native map and b) the false positives would be very different from random noise. Thus we argue that the filtering used in FT-COMAR based in common neighbours is not realistic and so the reported tolerance to noise could not be extended to real situations. In our case we have tested the robustness of the algorithm still against random noise (which in principle would have a different distribution than predicted false positives) but we do not perform any pre-filtering. We believe this to constitute a more realistic benchmark.

The tests performed here are based on randomly generated inaccurate contact maps which in principle differ significantly from ab-initio predictions. However from our results here we could conclude that with adequately precise ab-initio contact predictions one could produce reasonable models. In fact we applied successfully some of these ideas in the CASP8 community-wide experiment for structure prediction [37]. In that case we used template-based contact maps that led to 3D models comparable to those of established methods. The non-random noise of the template-based maps did not seem to affect significantly the 3D recovery.

## Conclusions

In this work we have studied the viability of computing 3D protein models from contact maps. We assessed the performance of a reconstruction procedure based on the well known distance geometry protocol used extensively in NMR protein structure determination.

We perform a comprehensive evaluation covering a representative set of the PDB spanning the 4 SCOP classes. We then explore several possible contact map definitions and evaluate the accuracy of the reconstructions based on RMSD to the available native structure.

We found that contacts based on the *C*_*β *_atoms are a better description of the 3-dimensional model than those based on *C*_*α*_, confirming other studies that used one-body and two-body empirical contact-based potentials for fold recognition to find this optimum. Reconstruction accuracy can be further improved by using the two contact definitions together *C*_*α *_+ *C*_*β*_.

With regards to contact cut-offs we found that the optimal lies in the region from 9 to 11Å. We do not observe, contrary to previous studies [26] that the accuracy improves for higher cut-offs. Because of the increasing amount of contacts that higher cut-off contact maps yield, we preferred as an optimal threshold the lower end of the optimal range. A contact map based on a 9Å cut-off achieves maximal geometrical information per contact.

Interestingly the accuracy of the reconstruction seems to be different for different classes of proteins. Particularly the all-*β *SCOP class yields very good accuracies across all its members as compare to the other classes, leading to the conclusion that some topologies are more amenable to be described in terms of single atom distance restraints.

These results are particularly valuable for the contact prediction community. As contact prediction ultimately aims at obtaining 3-dimensional models of protein structures the usage of our optimal contact definition findings should contribute to better accuracies of the predictions. At the same time the results can be useful in the structural alignment of proteins through contact map overlap [3]. These methods seek a 3D alignment by optimising a contact map overlap measure. Clearly contacts that contain better 3-dimensional information should lead to improved results in the final alignments.

Further our 3D recovery procedure seems to perform also very well even if only a partial subset of the contacts is available. With as little as 40% of the contacts reasonably good models can be produced. On the contrary the method is very sensible to the presence of non-real contacts. The introduction of restraints at random points in the chain is simply fatal for the recovery of the original structure. This indicates that contact predictions should focus on accuracy rather than coverage.

## Methods

### Reconstruction pipeline

This study is based on the TINKER molecular dynamics package [38], available at http://dasher.wustl.edu/tinker. In particular the *distgeom *[39] program was used for the generation of 3-dimensional protein models from distance restraints which is at the core of the contact map reconstruction procedure.

An interface to the TINKER package was developed (Java) providing a single command line executable as a one stop solution for contact map reconstruction, taking contact maps as input and outputting PDB files. The software is multiplatform (Linux, Windows and Mac) and only requires a working copy of the TINKER package locally installed.

We have made our program freely available under the terms of the GPL v.2 at http://www.molgen.mpg.de/~lappe/reconstruct.

### Reconstruction procedure

We generated distance restraints from the contact maps in the form of lower and upper bounds restraints for pairs of atoms (with standard value of 100.0 kcal/Å^2 ^for the force constant). The restraints were then fed into distgeom to generate a total of 30 models per structure using simulated annealing for refinement. The extensive study performed required a substantial amount of computation as we had 60 proteins, 3 contact-type definitions and 19 cutoff bins from 6 to 15 with 0.5 step. This gave a total of 3420 contact maps, for each of them we computed 30 structures in order to have a statistically meaningful sampling of the reconstruction space, resulting in a total of 102,600 models. The computations were carried out in a distributed fashion on a Linux cluster with over 100 CPUs.

The conformations found through the distance geometry protocol can not distinguish between the 2 enantiomers of the molecule, as chirality information is simply not present in the contact map. We overcome this problem by comparing to the native molecule through RMSD. The RMSD values for the conformation ensemble are found to be distributed bimodally, by simply choosing the lowest third of models as ranked by RMSD we are sure not to be falling into the wrong enantiomer.

### Contact maps and distance restraints

We used two definitions of contact maps in this study: *C*_*α *_and *C*_*β*_. Two atoms were considered to constitute a contact when their euclidean distances where below the given cut-off. In the *C*_*α *_model the backbone *C*_*α *_atom for each residue is chosen, whilst for the *C*_*β *_model the *C*_*β *_atom of the side chain of each residue is taken, except for Glycine where we use the *C*_*α *_atom.

For the reconstruction procedure we then need the contacts to be translated into distance restraints. Restraints were generated only for pairs of atoms corresponding to the contacts: *C*_*α *_atoms or *C*_*β *_atoms for each of the cases above. As upper bound of the restraint we used directly the distance cut-off, while for the lower bound value we used distance statistics derived from the PDB database. We proceeded by plotting the distance distribution for all *C*_*α *_or *C*_*β *_atoms and then choosing as our lower cutoff the value of the 90th percentile of the distribution.

### Distance Geometry

The distance geometry procedure in TINKER is an implementation of the established distance geometry algorithms used for NMR protein structure determination, see [20]. Crippen and Havel proposed the EMBED algorithm consisting of three steps: bounds smoothing, embedding and regularization (coordinate refinement). The bounds smoothing is the procedure by which the initial sparse set of distance restraints is extended to obtain a full set of distance ranges for all pairs of atoms. This is achieved by means of the triangle inequality starting from the distances of known pairs. Once distance restraints are found for all pairs one only needs to select at random a particular value from within the restraints. There are several strategies for this selection [40], the most effective one is metrization. To perform metrization one proceeds starting at a random atom, choosing distances for it and then readjusting the whole matrix through the triangle inequality procedure. By doing this for all atoms the result is a sampled distance matrix where the triangle inequality is fulfilled or in other words a metric matrix. Once we have a distance matrix of exact distances for all pair of atoms a very good approximation of the 3-dimensional embedding can be obtained through the 3 largest eigenvalues of a certain transformation of the distance matrix. The result of the embedding is a good solution to the given distance restraints, however the geometry of the molecule is still not good enough especially with regards to the bond distances and angles. Thus the need for a final regularization step consisting in the minimization of an error function of the restraint violations usually done through simulated annealing.

### Data set

In the selection of the data set we aimed at covering a diverse set of structures to ensure generality of the results obtained. We used a non-redundant PDB dataset of 60 proteins selected from SCOP release 1.73 [41]. Only monomeric, monodomain proteins from the four main SCOP classes and from highly populated folds are chosen. All proteins have resolutions better than 3.0Å, R-factor lower than 0.3 as well as no missing or ambiguous conformational data. A subset of 12 proteins, three per SCOP class, is selected from the dataset as used by Sathyapriya et al. [30]. From each group of 3 proteins, two fall in the size range of 100 - 120 amino acids and the third is three times as big as the other two. The PDB codes of the subset of proteins are given in Table 1.

## Authors' contributions

JD performed the bulk of the analysis, developed the software and drafted the manuscript. RS performed the analysis related to error tolerance of reconstruction. HS developed the software and participated in the design of the study. IF selected the protein subset and contributed drafting the manuscript. ML initiated the study and participated in its design. All authors read and approved the final manuscript.
